# Auxin-Cytokinin Balance Shapes Maize Root Architecture by Controlling Primary Root Elongation and Lateral Root Development

**DOI:** 10.3389/fpls.2022.836592

**Published:** 2022-04-25

**Authors:** M. Ángeles Rivas, Iván Friero, M. Victoria Alarcón, Julio Salguero

**Affiliations:** ^1^Departamento de Biología Vegetal, Ecología y Ciencias de la Tierra, Universidad de Extremadura, Badajoz, Spain; ^2^Departamento de Hortofruticultura, Instituto de Investigaciones Agrarias “La Orden-Valdesequera”, Centro de Investigaciones Científicas y Tecnológicas de Extremadura (CICYTEX), Junta de Extremadura, Badajoz, Spain

**Keywords:** lateral roots, auxin, cytokinin, maize, root system architecture

## Abstract

The root system is responsible for water and nutrients uptake from the soil, and therefore, its extension is basic for an efficient acquisition. The maize root system is formed by different types of roots, and the lateral root branching substantially increases the surface for nutrient uptake. Therefore, the regulation of lateral root formation is fundamental in the development of root functions. Root architecture is basically controlled by auxin and cytokinins, which antagonize in the formation of lateral roots (LR) along the primary root axis, with auxin, a stimulator, and cytokinins inhibitors of LR development. This interaction has been analyzed in several zones along the primary root where LRs in different developmental stages were located. The root has been divided into several zones, such as meristem, elongation zone, and mature zone, according to the developmental processes occurring in each one. As Arabidopsis root elongated more slowly than maize root, these zones are shorter, and its delimitation is more difficult. However, these zones have previously been delimitated clearly in maize, and therefore, they analyze the effect of exogenous hormones in several LR developmental stages. The inhibitory effect of cytokinin on lateral root formation was observed in already elongated primary root zones in which initial events to form new lateral roots are taking place. Contrarily, auxin increased LR formation in the primary root segments elongated in the presence of the hormone. The inhibitory effect of cytokinin was reversed by auxin in a concentration-dependent manner when both hormones were combined. However, auxin is unable to recover LR development in primary root zones that have been previously elongated only in the presence of cytokinin. This antagonistic auxin-cytokinin effect on LR development depended on the balance between both hormones, which controls the root system architecture and determines the formation of LR during the process of initiation.

## Introduction

The seedling root system is a basic organ in the earliest developmental stages of plants (Cuesta et al., 2013). Initially, this organ is the result of the primary root (PR) elongation and the recurrent branching along its main axis to form lateral roots (LRs), which are developed after germination and are major determinants of root system architecture (RSA) (Cho et al., 2019). In addition, other seminal roots complete the maize RSA (Hochholdinger and Tuberosa, 2009). The shape of the root system depends on the PR elongation and on the number and length of LRs formed and their subsequent growth. The plasticity of the plant growth developmental program provides the root with the ability to adapt its architecture to environmental conditions (Yu et al., 2019). Therefore, LRs form the main part of the RSA and are fundamental to optimize the ability for water and nutrient acquisition (Chang et al., 2015).

The processes of RSA formation are coordinated by plant hormones among auxins, and cytokinins are central molecules in the regulation of PR elongation together with LRs organogenesis (Bielach et al., 2012b; Dubrovsky and Forde, 2012). Auxins are considered as a key regulator of PR growth and LR formation (Alarcón et al., 2019). It is well-known that exogenous auxin inhibits PR elongation and increases LR density in maize roots (Han et al., 2012; Márquez et al., 2016) producing what is called stress-induced morphogenic response (Potters et al., 2007). Natural auxin indole-3-acetic acid (IAA) is synthetized in the shoot apex and transported basipetally to the base of the shoot; once in the root, IAA moves acropetally toward the root apex *via* the central cylinder and then basipetally to the elongation zones through the outer root tissues. The acropetal transport is involved in LR formation, whereas the basipetal transport regulates root elongation (Rashotte et al., 2001). The auxin transport is required for LR initiation as mutants in auxin transport show decreased LR formation (Benková et al., 2003). In addition, inhibition of IAA transport by the polar auxin transport inhibitor *N*-1-(naphthyl) thalamic acid (NPA) results in a reduction of LR that can be rescued if NAA is applied (Casimiro et al., 2001). Similar results have been reported in tobacco seedlings that increased first-order LR and auxin levels under drought stress. As 1-naphthaleneacetic acid (NAA) increased lateral root density (LRD) and the effects of drought stress were abolished by NPA, it has been proposed that IAA polar transport is also required for LR initiation (Wang et al., 2018). Moreover, mutants that overproduce IAA also exhibit higher LRD, whereas mutants with low IAA levels have decreased numbers of LRs (Boerjan et al., 1995).

Auxin has a dominant role in the specification of founder cells (Dubrovsky et al., 2008) and in the initial stages of LR development (Marhavý et al., 2013). An accumulation of auxin precedes founder cell specification and LR initiation (De Smet et al., 2006; De Rybel et al., 2010). This accumulation is dependent on the polar transport of auxin mediated by the auxin efflux carrier PIN (Benková et al., 2003).

Cytokinins are also involved in RSA. In *Arabidopsis*, cytokinins reduce the cell elongation and consequently the root elongation (Beemster and Baskin, 2000). Exogenous cytokinins applied at high concentrations reduce PR elongation in maize, but contrarily to auxin, also decreased LR formation (Márquez et al., 2019). In *Arabidopsis*, cytokinins constrain both LR initiation and development (Li et al., 2006; Fukaki and Tasaka, 2009), and supraoptimal cytokinin inhibits the rice seminal root growth (Zou et al., 2018). Mutants that overproduce cytokinins exhibit decreased LR density (Laplaze et al., 2007), whereas transgenic plants with reduced levels of cytokinins enhanced LR formation (Werner et al., 2003; Lohar et al., 2004). In addition, the response to genotoxic stress that results in decreased LRD is mediated by an increase in cytokinin levels (Davies et al., 2016). On the contrary, it has been proposed that cytokinin inhibition includes first division in pericycle founder cells and in the outgrowth of young primordia (Dubrovsky et al., 2008; Chang et al., 2013).

Auxin and cytokinins interact to regulate plant growth and developmental processes (Schaller et al., 2015; Kurepa et al., 2018). LR development has been largely reported to be controlled by auxin and cytokinins acting as a stimulator and inhibitors, respectively (Jing and Strader, 2019). The antagonistic actions of auxin and cytokinins in LRs initiation and elongation have been largely reported (Bielach et al., 2012a; Singh et al., 2015). Additionally, it has been proposed that auxin transport inhibition by cytokinins decreases auxin levels in root and consequently reduces LR formation (Shkolnik-Inbar and Bar-Zvi, 2010). However, auxin inhibits LR elongation once lateral root primordium (LRPs) has emerged (Márquez et al., 2016). Auxin is unable to reverse this inhibitory effect by cytokinins on LR formation. Increased levels of cytokinins by exogenous application result in the reduction of LR density, whereas a decrease in the cytokinin activity enhances LR organogenesis (Li et al., 2006). Both auxin application and root tip removal stimulate LR formation, and this effect was prevented by cytokinins. However, the inhibition by benzylaminopurine (BAP) could not be overcome by indole-3-butyric acid (IBA). BAP inhibits and IBA induces LR formation; but, the LRPs induced by IBA would not emerge when roots were grown in a medium with BAP (Zhang and Hansenstein, 1999). It has been suggested that cytokinin inhibition on LR initiation depends on a specific loss of response to auxin in founder cells (Li et al., 2009). Cytokinins have been demonstrated to control the auxin flow in plant organogenesis (Marhavý et al., 2014), alter the expression of PIN genes in LR founder cells, and prevent auxin accumulation required to initiate LRP formation (Laplaze et al., 2007). In addition, it has also been proposed that the inhibitory cytokinin action on LRP development depends on the robustness and stability of the auxin gradient and that LR initiates a zone with elevated levels of biological active cytokinins but repressed cytokinin responses (Bielach et al., 2012a). On the contrary, auxin is a negative regulator of cytokinin levels (Nordström et al., 2004), whereas cytokinins interfere with the auxin polar transport (Ruzicka et al., 2009) and negatively affect LR development by impinging on PIN-dependent auxin transport (Marhavý et al., 2011; Moreira et al., 2013). Lateral root spacing in *Arabidopsis* is regulated by cytokinins, and a model proposes that during LR initiation a local cytokinin signal is generated in the neighboring pericycle cells inhibiting LR initiation of other LRPs, thus acting as a paracrine hormone (Chang et al., 2015).

The aim of this work was to study the antagonistic interaction between auxin and cytokinins on LR initiation and development in maize by analyzing the action of each hormone at several levels of the LR development. This interaction has been performed in several zones along the primary root where LRs in different developmental stages were located. The root has been divided into several zones, such as meristem, elongation zone, and mature zone, according to the developmental processes occurring in each one. As Arabidopsis root elongated more slowly than maize root, these zones are shorter and its delimitation is more difficult.

As expected, auxin application clearly stimulated LR formation and cytokinins application inhibited LR formation. The results obtained in combined treatments suggested that LR development depends on the auxin-cytokinin balance. In addition, the developmental stage of LR is a crucial point. The application of cytokinins reduced LRD in root zones in which the first events to LR formation are taking place, and auxin was unable to reverse this effect. Nevertheless, the stimulatory effect of auxin was observed mainly in the root segment elongated in the presence of this hormone. Moreover, auxin was able to reverse the inhibitory effect of cytokinins on LR development in root zones elongated in the presence of both hormones if the balance is favorable to auxin. These results suggested that cytokinins exert their inhibitory effect mainly in the priming of founder cells and LR initiation.

## Results

The inhibitory effect of cytokinins and auxin on PR elongation was analyzed by adding several concentrations of 6-furfurilaminopurine (KIN) and NAA to the hydroponic growth medium where roots were growing. Root elongation was estimated by measuring the PR length several times before and after the hormone application. Control roots of 80–90 mm length elongated at 3.29 ± 0.15 mm/h during 48 h, and the application of both KIN (0.001–0.1 μM) and NAA (0.001–0.05 μM) reduced the concentration of PR elongation dependently (Table 1).

**TABLE 1:** Primary root elongation and lateral root density (LRD) in proximal region after 48 h of treatment with several concentrations μM of KIN and NAA.

		Lateral root density [(LR+PRL)cm^–1^]											
		Proximal region						Distal region					
		Distance to root apex when treatment was performed (cm)						Distance to root apex when treatment was performed (cm)					
Treatment	Elongation [mm/48 h]	−5.5	−4.5	−3.5	−2.5	−1.5	−0.5	0.5	1.5	2.5	3.5	4.5	5.5
CTR	170 a	28 a	28 a	20 a	18 a	16 a	12 b	13 b	14 b	11 b	15 b	14 b	14 a
0.001 KIN	167 a	28 a	22 a	20 a	19 a	14 a	10 b	13 b	11 c	12 b	12 b	11 b	10 b
0.005 KIN	158 a	30 a	23 a	21 a	17 a	6 b	2 c	8 c	7 d	7 d	8 c	7 c	7 c
0.01KIN	142 b	28 a	23 a	20 a	16 a	6 b	0 c	4 d	3 e	5 d	4 d	3 d	4 d
0.1 KIN	34 f	27 a	22 a	20 a	13 b	4 b	0 c	0 e	1 e	2 e	0 e	0 e	0 e
0.001 NAA	153 a	33 a	30 a	24 a	20 a	17 a	10 b	14 ab	14 b	12 b	14 b	14 b	14 a
0.005 NAA	138 b	33 a	27 a	22 a	17 a	15 a	10 b	16 ab	16 ab	16 ab	17 a	17 a	14 a
0.01 NAA	104 d	26 a	23 a	18 a	16 a	15 a	12 b	20 a	20 a	19 a	18 a	18 a	16 a
0.05 NAA	36 f	30 a	22 a	18 a	16 a	17 a	15 a	15 ab	10 c	1 e	0 e	0 e	0 e
0.01 NAA + 0.001 KIN	117 c	24 a	22 a	18 a	18 a	14 a	12 b	17 ab	17 ab	18 a	18 a	17 a	15 a
0.01 NAA + 0.005 KIN	107 d	29 a	22 a	20 a	18 a	10 b*	3 c*	13 b*	14 b*	14 b*	14 b*	14 b*	12 b*
0.01 NAA + 0.01 KIN	66 e	27 a	24 a	20 a	16 a	4 b*	0 c*	9 c*	8 d*	10 c*	9 c*	7 c*	1 e*

*The point 0 was the root apex when treatment was performed. The proximal and distal regions have been divided in six 1 cm-segments and the values of LRD in each segment were assigned to the medial point. Different letters in elongation indicate significant differences; in LRD different letters indicate significant differences in the same segment (ANOVA and Tukey’s test P < 0.05); n = 20. Asterisks in combined treatments indicate significant differences compared to NAA at 0.01 μM applied alone (Student’s test P < 0.05).*

**FIGURE 1:**
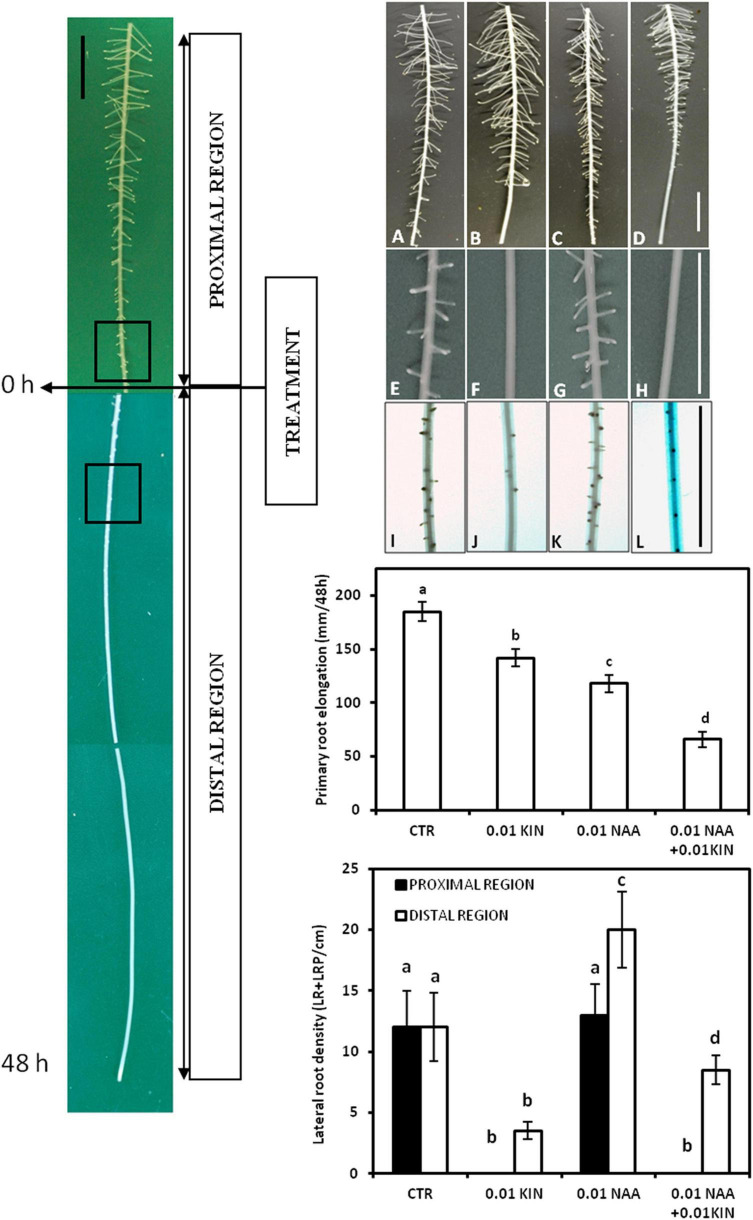
Effects of auxin and cytokinin on lateral root development. Experimental design of treatments: hormone application was performed in roots of 80–90 mm in length, and after 48 h, two regions, proximal and distal, were formed. The effect of NAA and KIN on lateral root density (LRD) was analyzed in the most sensitive segments in both regions. These segments are indicated in the figure by the squares in the proximal and distal regions. Both KIN and NAA were applied at 0.01 μM. Seedlings were grown for 48 h after treatment, and then, segments corresponding to the zones elongated before (proximal region) and after (distal region) were photographed. Panels **(A–D)** correspond to 6 cm of the proximal region, panels **(E–H)** correspond to the 1-cm segment located in the most apical zone of the proximal region, and panels **(I–L)** correspond to the 1-cm segment located between the 2 and 3 cms of the distal region. **(A–I)** (control roots); **(B–J)** (0.01 μM KIN-treated roots); **(C–K)** (0.01 μM NAA-treated roots); **(D–L)** (0.01 μM KIN + 0.01 μM NAA-treated roots). Primary root elongation in mm within 48 h after treatment was represented, and this figure shows the inhibitory effect of both auxin and cytokinin. Lateral root density in the most sensitive 1-cm segments of both proximal and distal regions grown before and after treatment are also shown in the figure. Data represent mean ± standard deviation (*n* = 20 roots). Different letters indicate significant differences in elongation and lateral root density (ANOVA followed by Tukey’s test at *P* < 0.05). Bars represent 1 cm in all the figures.

The application of KIN and NAA to the growth medium generated two segments in the PR, namely, proximal and distal regions (Figure 1), which have elongated before and after the treatment, respectively (Márquez et al., 2016). Moreover, the proximal region has also been divided into two zones, namely, basal and apical (Márquez et al., 2019), based on the presence or absence of LRP, respectively, when the treatment was performed. In these roots, just before treatment, LRPs in the most initial stages were detected at 26.5 ± 2.8 mm from the root apex. After 48 h of treatment, LRs were observed in the proximal region, whereas in the distal region, short LRs less than 1 mm in length and mainly LRPs were detected. As expected, the longest LRs were located in the basal zone of the proximal region after 48 h of treatment (Márquez et al., 2016). This occurs because LRs were formed in a sequence apparently acropetal and LRD decreases from basal to apical regions of the proximal zone (Figure 1).

In this study, we have analyzed the effect of KIN and NAA in both proximal and distal regions separately by LR quantification 48 h after the application of auxin and cytokinin (Dubrovsky et al., 2006). In the proximal region, KIN significantly reduced the lateral root density (LRD) in the 2 apical cm at concentrations up to 0.005 μM (Table 1 and Figure 1). This inhibition was higher in the most apical 1-cm segment where LRD is reduced to values near to 0 when KIN was applied at 0.01–0.1 μM, indicating the total inhibition of LR formation. On the contrary, NAA did not affect LRD significantly in the entire proximal region except in the most apical cm (Table 1). Moreover, neither the inhibitory effect of KIN nor the stimulatory action of NAA on LR formation was observed in the basal zone of the proximal region, but no significant differences in LRD between KIN- or NAA-treated and control roots were found. In the distal zone, KIN reduced LRD in a concentration-dependent manner. This dependency is observed along 6 cm of the distal segment grown under the presence of KIN (Table 1 and Figure 1). In contrast, NAA increased LRD in the same segment as a consequence of the stimulatory effect of auxin in LR formation (Table 1). The photographs in Figure 1 provide us more information about the effect of KIN and NAA and LR development. The inhibitory effect of KIN (0.01 μM) on LRD in the most apical zone in the proximal region was particularly clear, since no LRPs were detected there. However, the last LR observed at about 1.2 cm from the end of this proximal region is about 2 mm length (Figures 1B,F). In the distal zone, 0.01 KIN strongly reduced LRD (Figure 1J). The stimulatory effect of NAA and the inhibitory action of KIN regarding LRD in the distal region can be observed simply by comparing the number of LRPs and LRs between several treatments (Table 1 and Figure 1). In addition, in the distal region, emerged LR was only observed in untreated and 0.01 μM NAA-treated roots, whereas in treatment with KIN at 0.01 μM, only LRPs were visible. Therefore, KIN not only delays LRP development but also causes a reduction in the LRP initiation, as few LRPs were observed (Figure 1F).

To study the antagonistic action between cytokinins and auxin, combined treatment of these two hormones was applied. As 0.01 μM NAA is the most effective concentration in stimulating LR formation in the distal region, we have combined this concentration with three levels of KIN: 0.001, 0.005, and 0.01 μM. The result presented in Table 1 showed that 0.001 μM KIN, which did not reduce LRD with respect to those of untreated root, did not diminish the stimulatory effect of 0.01 μM NAA (Table 1). KIN at 0.005 μM caused a reduction of 50% in average in LRD (Table 1). However, 0.01 μM NAA reversed this inhibitory effect by 0.005 μM KIN effect as the combined treatment showed LRD values higher than those measured with 0.005 μM KIN alone.

Finally, 0.01 μM KIN strongly reduced LRD from 12.8 LRs/cm (untreated root) to 3.6 LRs/cm expressed as an average in the distal zone (Figure 1). In the combined treatment (0.01 μM KIN + 0.01 μM NAA), we observed a partial LRD recovery that showed a mean value of 7.3 LRs/cm. Taken together, these results indicated that the antagonistic interaction of auxin-cytokinin on LR formation is dependent on the concentration. The photographs shown in Figure 1 supported these results; 0.01 μM NAA only slightly increased LRD in the apical zone of the proximal region but reduced LR length along the entire proximal region. KIN at 0.01 μM reduced LRD at the most apical cm of the proximal region but did not reduce LR length. In the combined treatment, NAA did not reverse the inhibitory effect of KIN on LRD but decreased the LR length. In the apical zone, only LRPs were detected, whereas in the control, emerged LRs were observed (Figure 1).

In the distal region, the stimulation by auxin is clear as well as the inhibitory effect of cytokinin. However, in the combined treatment (0.01 μM NAA + 0.01 μM KIN), we observed that auxin only partially reversed the inhibitory effect of kinetin in LRD (Figure 1).

**FIGURE 2:**
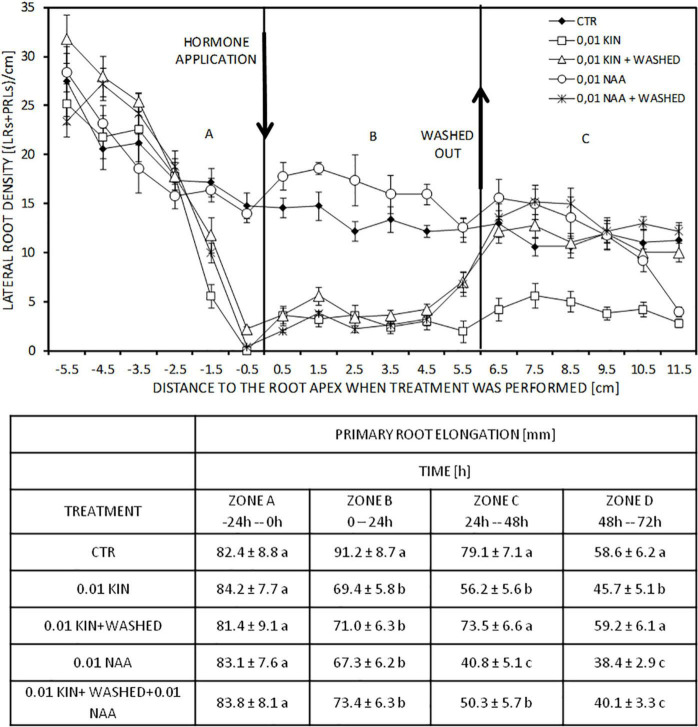
Effect of KIN withdrawal on PR elongation. Roots of 80–90 mm length were incubated with 0.01 μM KIN for 24 h. Then, roots were washed and transferred to a new medium KIN-free or with 0.01 μM NAA for the next 48 h. To analyze differences, appropriate controls were used. The experiment generates three zones: (A) proximal region; (B) distal region; and (C), regions elongated after withdrawal. LRDs (LRD = LRs + LRPs) were quantified in the three zones: (A) proximal region; (B) distal region; and (C) region elongated after withdrawal. After withdrawal, roots were grown for two more periods of 24 h producing zones C and D, respectively. LR density was only quantified in zone C but not in zone D. Data represent mean ± standard deviation (*n* = 10). Table indicates the PR elongation at different periods. Data represent mean ± standard deviation (*n* = 20 roots). Different letters indicate significant differences in the same period (ANOVA followed by Tukey’s test at *P* < 0.05).

Root elongation inhibition by cytokinin required the continuous presence of the hormone in the growth medium, and its withdrawal produced a recovery on PR elongation rate (Figure 2 bottom). Roots of 80–90 mm in length elongated 91 mm, whereas the application of 0.01 μM KIN or 0.01 μM NAA reduced this elongation to about 70 mm in 24 h (Zone B). In the subsequent periods of 24 and 48 h, KIN and NAA inhibited PR elongation compared with control roots (Zone D and D). When, after incubation with KIN for 24 h, KIN was removed, and the PR elongation rate recovered. As expected, the application of auxin, after cytokinin-withdrawal, inhibited root elongation; root growing in free regulator medium after cytokinin withdrawal has grown 132 mm in 48 h, whereas it elongated only 90 mm in the presence of 0.01 μM NAA (Figure 2 bottom). Regarding LR formation, to know which root zones are affected by exogenous cytokinins, roots were first treated with KIN and then washed and transferred to a new regulator-free or supplemented with auxin media. This experiment provided us with several segments of PR grown in different conditions: (a) a segment corresponding to the proximal region elongated in the absence of cytokinin, but in which LRs have developed under the presence of KIN (Zone A); (b) a segment elongated under cytokinin; in this case, both elongation and LR development took place in the presence of cytokinin (Zone B); and (c) a segment grown after transference to a new medium without cytokinin, in which auxin can be added (Zone C) (Figure 2, top). In the most apical 2 cm of the proximal region (Zone A), KIN at 0.01 μM drastically reduced LRD to values near to 0, but 0.01 μM NAA did not change LRD. In the segment grown after the application of NAA and KIN (Zone B), the stimulatory effect of auxin and the inhibitory effect of cytokinin in LRD were clearly observed. Untreated roots presented an LRD average in homologous segments elongated at the same time of 13.2 LRs/cm; 0.01 μM NAA increased LRD to 16.6 LRs/cm, whereas 0.01 μM KIN reduced LRD to 3.1 LRs/cm. After cytokinin withdrawal (Zone C), LRD is recovered immediately, and even more apical segments grown in the presence of KIN (Zone B) showed an increase in LRD. In this segment grown after withdrawal, LRD in untreated roots (11.3 LRs/cm) and KIN-treated roots after replacing the cytokinin medium with basal medium (11.4 LRs/cm) were similar. Moreover, LRD of NAA-treated roots for 72 h and LRD of NAA-treated roots after KIN withdrawal are similar in the 3-cm segment more close to those grown in the previous 48 h; the values were 14.7 and 14.6 LRs/cm, respectively. However, roots incubated for 72 h with NAA 0.01 μM showed a reduced length (134 mm vs 215 in control roots) and LRD decreased rapidly in segments near the root apex. This decline of LRD was observed at 40 mm away from the root apex as that usually occurs in untreated roots.

**FIGURE 3:**
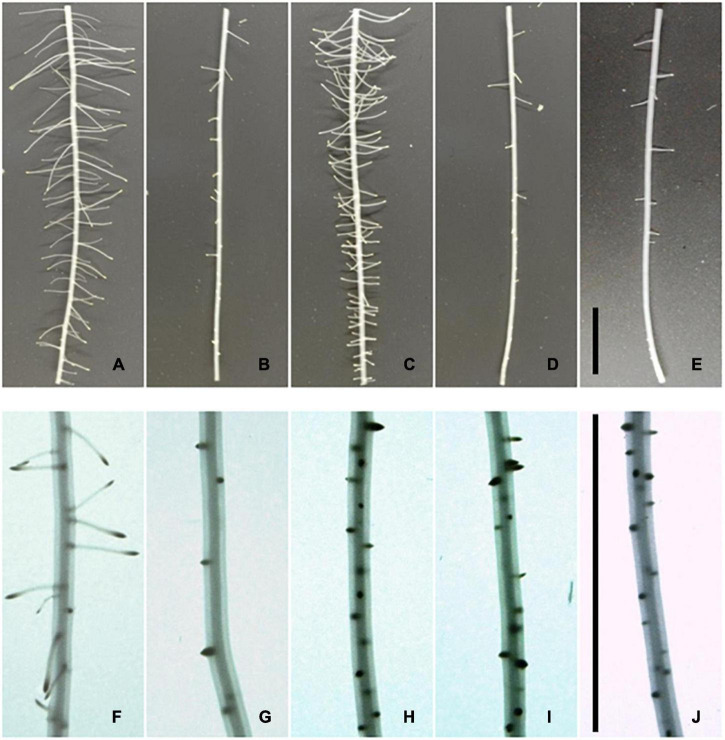
Effect of 0.01 μM KIN in the root undergone to withdrawal on lateral root density. Photographs correspond to the distal region (6 cm) grown in the presence of regulators **(B)** and to the 1-cm segment located between 2 and 3 cm in the zone elongated after withdrawal **(C)** in Figure 2. Growth conditions are indicated in the figure: **(A,F)** (control roots); **(B,G)** 0.01 μM KIN; **(C,H)** 0.01 μM NAA; **(D)** distal region grown 0.01 μM KIN; and **(I)** segment of the region **(C)** elongated after the root had undergone withdrawal; **(E)** distal region grown in the presence of 0.01 μM KIN; and **(J)** segment of the region **(C)** elongated after withdrawal supplemented with 0.01 μM NAA. Note that LRs were only observed in the distal region, but only LRP was observed in the region elongated after the root had undergone withdrawal, except the control roots.

Figure 3 presents homologous zones of several treatments from Figure 2. The photographs show the distal region elongated after hormone addition and the region elongated after withdrawal designated as zone B and zone C, respectively, in Figure 2. LRs developed in untreated and NAA-treated roots along the entire zone B, but a strong reduction was observed in treatments with KIN (Figures 3A–C). Although LRD was diminished by KIN, LRs had elongated and LRPs were not observed in zone B (Figure 3B). However, in the roots that have undergone withdrawal, a considerable number of LRPs were observed in the most apical part of this region independently of NAA being added after withdrawal (Figures 3D,E). In the region elongated after withdrawal (zone C), stimulation of LRD by NAA was observed, and the reduction in LRD by KIN was observed if it was not withdrawn (Figures 3G,H). After withdrawal, the region developed without KIN (Figure 3I) showed increased LRD compared with region B developed in the presence of the hormone (Figure 3D).

## Discussion

The exogenous application of auxin and cytokinin inhibited PR elongation (Table 1). Cytokinins have been largely reported to inhibit root growth in dicotyledonous (Werner et al., 2003; Delle Ioio et al., 2007) and monocotyledonous plants (Zou et al., 2018; Márquez et al., 2019). The inhibitory effect of auxins in root elongation is also well-known (Martínez de la Cruz et al., 2015; Márquez et al., 2016). This inhibitory effect depended on the applied concentration for both regulators. As the PR branching requires that PR provides new zones for LRs to develop (Bielach et al., 2012b), PR elongation is necessary. In this study, the concentration of KIN only slightly reduced PR elongation and allowed the quantification of LRPs in the segment of PR root developed after KIN treatment.

The hormone addition to the growth medium forms in the PR two contiguous segments called proximal and distal regions that have elongated before and after treatment, respectively. After 48 h of treatment, the proximal region exhibited mainly LRs but the younger distal region developed only LRPs (Márquez et al., 2019), indicating that LRPs initiated in an apparent acropetal sequence by reactivation of cell proliferation in a group of founder pericycle cells (Alarcón et al., 2016). In order to analyze the effect of the presence of the hormone in PR elongation on the subsequent LR formation, LRD was presented separately in the proximal and distal regions (Table 1). While cytokinin has an inhibitory effect, auxin increased LRD. These effects are produced in a concentration-dependent manner (Table 1). As it has previously been shown, the degree of inhibition on LR formation differed along the PR axis according to the several stages of the LRPs (Márquez et al., 2019). In our experiments, PR at the moment of hormone application has been divided into two zones according to the visualization of LRPs. As reported already (Márquez et al., 2019), we observed that cytokinin inhibited lateral root formation in zones where LRPs are in the earliest phases of initiation. However, in more basal zones of the proximal region, no inhibition by cytokinin was found. These results agree with those observed in rice, where cytokinins do not affect LR emergence once LRPs have been formed. The LRPs that have developed in the cytokinin-free medium can emerge normally in the solution containing an inhibitory concentration of kinetin that inhibits the LRP formation (Rani et al., 2005). However, in maize, the effect of kinetin on LR formation was different in segments elongated in the presence or absence of KIN. LR formation was strongly inhibited in zones where LR initiation took place, but no effect has been observed in the same roots in the zones with LRP developed before KIN application (Figure 2 and Table 1). However, auxin did not strongly modify LRD in the proximal zone. Only high NAA concentrations slightly increased LRD in the apical zone of this region (Table 1).

In the entire distal region, cytokinin reduced the LRD in a concentration-dependent manner (Table 1). However, we observed that concentrations that had totally inhibited LR development in the proximal region only partially inhibited LRD in this distal region. This result indicated that hormonal conditions of PR elongation affect future LRP formation and suggest a root capability of adaptation to hormonal level in accordance with previous reports (Márquez et al., 2019). Contrarily, NAA increased LRD along the distal region. LRD (Table 1).

Taken together, these results indicate that auxin and cytokinins have antagonistic actions in LRP formation in the early stages that correspond with priming of pericycle founder cells located in the apical zone of the proximal region. However, when new segments were formed by cell division and elongation in the presence of increased cytokinin levels, the sensitivity to the hormone diminished, and consequently, cytokinins were unable to inhibit the LRP initiation completely. Cytokinin application reduced LRD in the apical segment of the proximal region, whereas auxin had only a slightly significant effect at very high concentrations (Table 1). In this segment, only emerged LRs were observed, but no LRPs were detected after 48 h of treatment (Figure 1B). This indicates that after 48 h, the already formed LRPs were able to develop LRs, but no new LRPs were formed. Moreover, in the proximal region, the lengths of LRs in the KIN-treated roots were similar or even longer than the control roots. Nevertheless, no LRPs were detected in more apical zones (Figure 1B) where LRPs were not visible in the earliest stages of the LRP initiation when cytokinin was applied 48 h before. This indicates that LRPs formed before the cytokinin was applied were able to develop and emerge as LRs but no new LRP initiation events took place in the presence of cytokinin.

The antagonistic action of auxin and cytokinins is well-known (Ruzicka et al., 2009; Bishopp et al., 2011), but few information about LR formation by combining both regulators has been reported (Bielach et al., 2012a,^2017^). In this study, the development of LR has been deeply analyzed by combining the most stimulating NAA concentration on LR formation with three levels of KIN. The antagonism of these hormones on LR formation is clearly demonstrated to be dependent on concentration as NAA reversed the inhibitory effect of KIN in the function of the concentration of KIN applied (Table 1). According to these results, it has been concluded recently that auxin inhibits the response to low concentrations of cytokinins, but it is ineffective when the cytokinin 6-bencylaminopurine (BA) is applied in a higher concentration (Kurepa et al., 2018).

The inhibition of PR elongation required the presence of cytokinin, but after withdrawal PR recovered the elongation rate (Figure 2) reaching values similar to those of control roots. The withdrawal performance produced three zones which showed different characteristics as explained previously (Márquez et al., 2019). The LRD in these zones provided information about the persistence of the kinetin effect. After KIN withdrawal, LRD values were recovered (Figure 2), thus indicating the necessity of the continuous presence of KIN for the inhibitory effect in LRP formation.

Regarding the effect of hormones on LR elongation, we observed that in the presence of KIN, only LR was developed and no LRPs were detected in next zones. This is a new support for the hypothesis that KIN inhibits LRP initiation rather than emergence and LR elongation. This result is in accordance with results in Arabidopsis, which have shown that exogenous cytokinins have no inhibitory effect on the development of initiated LRPs (Li et al., 2006).

Finally, we found that LR elongation is less sensitive to the inhibition by cytokinins than initiation. Cytokinin inhibition of LRs is restricted to the time when founder cells are initiating LRPs. Therefore, the initiation of LRPs in founder cells is confined to a certain space and time. When hormonal conditions do not permit LRP initiation, pericycle founder cells lose the capability to initiate LRPs, and this ability is not recovered by root when hormonal conditions became favorable. These results agree with the spatiotemporal regulation of LR organogenesis by cytokinins (Bielach et al., 2012b).

The inhibitory effect of cytokinins is different in the apical zone of the proximal region than those in the distal region. KIN completely inhibits LR in the apical zone of the proximal region, but LRP formation is recovered in the segment of the distal region elongated after cytokinin addition. These results could be explained by the robustness of the auxin gradient. It has been proposed that the cytokinin activity during the early phases strongly interacts with the auxin gradient formation, whereas in more basal regions of the root where LRs are in more advanced stages, cytokinins could not affect the auxin gradient (Bielach et al., 2012a), and therefore, the inhibitory effect of cytokinins should be located in zones with LRPs in the earliest stages of development. In rice, cytokinins inhibit LR initiation but it does not inhibit LR emergence once LRPs are formed (Rani et al., 2005). However, studies in *Arabidopsis* suggest that cytokinins affect both the first formative cell division of pericycle founder cell and the subsequent outgrowth of LRPS to produce new lateral roots (Chang et al., 2013).

It has been proposed that the robustness of the auxin gradient determines the effect of cytokinins (Bielach et al., 2012a). The auxin in the root is transported from the root base to the apex contrarily to cytokinins synthesized in the root apex and transported acropetally in the root. Then, the auxin gradient declines in most apical regions, and consequently, its robustness decreases. Therefore, exogenous cytokinins would generate more disturbances in the auxin gradient in more apical zones, and consequently, the inhibition of LRP formation increased. This is in accordance with observations showing that cytokinins have a higher inhibitory action before morphologically visible events take place. More evidence was found in mutants with reduced cytokinin content which showed an increase in LRP density in the first stage of LRP development (Chang et al., 2013). On the contrary, it has been shown that auxin promotes the transcription of AHP6, a repressor of the cytokinin signaling (Bishopp et al., 2011). This cytokinin repressor AHP6 is involved in the appropriate localization of PIN1, which directs the auxin flow to accumulation in founder cells and initiates the first asymmetrical cell divisions (Moreira et al., 2013).

In summary, the results of combined treatments with cytokinin and auxin on LR development in maize showed an antagonistic action between both hormones. It is clearly illustrated that root system architecture is controlled by auxin-cytokinin interaction and that the LR formation depends on the balance between both hormones. Indeed, this balance determines the RSA by controlling the process of LR initiation.

## Materials and Methods

### Plant Material and Growth Conditions

The experimental design in this work essentially follows the methodology reported in previous works (Márquez et al., 2019). The seeds of *Zea mays* L. cv. DK 6980 were washed three times and immersed in distilled water with aeration at 30^°^C. After 24 h, the seeds have developed radicles of about 1 mm in length. The seeds were placed in plastic containers on filter paper soaked with distilled water. The seeds were covered with filter paper and grown in darkness at 30^°^C. They were kept vertically for 24 h until their roots reached a length of 30 ± 5 mm. Disks with 10 selected seedlings of uniform root length were placed in a growth medium composed of 1 mM HEPES, 1 mM CaCl_2_, and 10 mM KCl, pH 6.0, and grown at 30^°^C in darkness in bottles of 1.35 L. The growth medium was aerated using an aquarium pump. After 24 h, the PRs were 80–90 mm long, and treatments were performed by adding small volumes of concentrated solutions of NAA and KIN to the growth medium. Cytokinin (6-furfurilaminepurine, KIN) was dissolved in 1 N NaOH and then diluted in 1 mM HEPES buffer to make a 0.1 mM stock solution. Auxin (1-naphthaleneacetic acid, NAA) was dissolved in HEPES to 0.1 mM stock solution. The volumes added to the growth medium were less than 0.1% of the total volume. All the chemicals were supplied by Sigma-Aldrich except CaCl_2_ and KCl (Merck). Before application, the root lengths were individually measured and further measurements were taken 24 and 48 h after applying the phytohormones using a ruler (accuracy ± 1 mm). After the addition of hormones, two consecutive root regions were established corresponding to the segments elongated before and after the treatment. The proximal region (about 6 cm), which grew up before the auxin application, could be subdivided into two different zones: a basal zone that corresponded to the oldest segment exhibiting primordia of lateral roots (LRP) when the hormone was applied, and an apical zone without any detectable LRP when hormones were applied. The root portion that elongated within 24/48 h after the treatment was denominated as the distal region (Márquez et al., 2016, 2019). In experiments where roots were transferred from a medium with cytokinin to a new hormone-free growth medium, the hormone was eliminated by submerging the roots two times in 1 mM HEPES and 1 mM CaCl_2_ before transferring to the new medium.

### Root Growth and Lateral Root Quantification

The roots were grown for 48 h to allow the development of LRPs to LRs and thus facilitate LR quantification after treatment. The primary root length was then measured, and several root zones were selected. These zones were then fixed in FAA [50% (v/v) ethanol + 36% (m/v) formaldehyde + 100% acetic acid, 91:6:3] for 48 h. After that, the roots were kept in 70% ethanol for 48 h. Both the proximal and distal regions were photographed along the entire root length in 1-cm segments. Several 1-cm long root segments were photographed. Then, LRs and LRPs were counted in each segment using the ImageJ program. LR density (LRD) was expressed as the number of LRs and LRPs per cm.

In the distal region where LRs are not usually observed, LRPs were counted under a dissecting microscope. Since roots were grown for more than 48 h after treatment, LRPs had developed sufficiently and were easily detected. In this work, the developmental stages of LRPs have not been studied, so a histological study has not been carried out as reported in other studies in maize (Husakova et al., 2013).

### Statistical Analysis

Data represent means ± standard deviation of 20 seedlings per treatment. Since experiments were performed in containers with 10 seedlings, roots from at least two containers were measured for each treatment. Experiments were repeated at least twice independently. Comparison between the media was performed, after Kolmogorov-Smirnov’s test for normality, by Student’s test or ANOVA followed by Tukey’s test at *P* < 0.05 using SPSS v 21.0. Statistics for LRD between different treatments was performed by comparing the LRD in comparable zones in different plants under different treatments.

## Data Availability Statement

The raw data supporting the conclusions of this article will be made available by the authors, without undue reservation.

## Author Contributions

MVA and JS conceived and designed the research and analyzed the data. MÁR and IF performed the experiments. JS wrote the manuscript. All authors read and approved the manuscript.

## Conflict of Interest

The authors declare that the research was conducted in the absence of any commercial or financial relationships that could be construed as a potential conflict of interest.

## Publisher’s Note

All claims expressed in this article are solely those of the authors and do not necessarily represent those of their affiliated organizations, or those of the publisher, the editors and the reviewers. Any product that may be evaluated in this article, or claim that may be made by its manufacturer, is not guaranteed or endorsed by the publisher.
